# Microbial bioinformatics 2020

**DOI:** 10.1111/1751-7915.12389

**Published:** 2016-07-29

**Authors:** Mark J. Pallen

**Affiliations:** ^1^ Microbiology and Infection UnitWarwick Medical SchoolUniversity of WarwickCoventryCV4 7ALUK

## Abstract

Microbial bioinformatics in 2020 will remain a vibrant, creative discipline, adding value to the ever‐growing flood of new sequence data, while embracing novel technologies and fresh approaches. Databases and search strategies will struggle to cope and manual curation will not be sustainable during the scale‐up to the million‐microbial‐genome era. Microbial taxonomy will have to adapt to a situation in which most microorganisms are discovered and characterised through the analysis of sequences. Genome sequencing will become a routine approach in clinical and research laboratories, with fresh demands for interpretable user‐friendly outputs. The “internet of things” will penetrate healthcare systems, so that even a piece of hospital plumbing might have its own IP address that can be integrated with pathogen genome sequences. Microbiome mania will continue, but the tide will turn from molecular barcoding towards metagenomics. Crowd‐sourced analyses will collide with cloud computing, but eternal vigilance will be the price of preventing the misinterpretation and overselling of microbial sequence data. Output from hand‐held sequencers will be analysed on mobile devices. Open‐source training materials will address the need for the development of a skilled labour force. As we boldly go into the third decade of the twenty‐first century, microbial sequence space will remain the final frontier!

Where will microbial bioinformatics be in 2020? Well, let us start by looking back. The last two decades have seen astounding progress in our ability to sequence microbial genomes (Loman and Pallen, 2015). Microbial bioinformatics has by and large kept pace with the resulting data deluge, now clearly emerging as distinctive discipline in its own right, driven forward by an enthusiastic global community of dedicated microbial bioinformaticians (Loman and Watson, 2013). We can expect this community to continue to grow in the coming years, as microbiologists across the world grapple with established and emerging challenges, including antimicrobial resistance, microbial biodiversity, understanding microbial communities and their genes (microbiomes), synthetic biology and the adoption of genome sequencing as a routine approach in the clinical and research laboratories (Cameron *et al*., 2014; Koser *et al*., 2014; Brown *et al*., 2015; Luheshi *et al*., 2015; Shanahan, 2015).

It is worth stressing that harnessing bioinformatics to the study of microbial genes, genomes and metagenomes clearly does provide a distinctive challenge – rather than taking aim at the fixed, relatively tractable target of a human, animal or plant genome, instead, here, we have to deal with genomic information derived from thousands of microbial pathogens, millions of commensal species and as many as a billion environmental microbial species (Locey and Lennon, 2016): a distributed and dynamic system of countless billions of genes, many orders of magnitude larger than the human gene set. The resulting deluge of sequence data plainly brings the problems of big data to microbial bioinformatics (Eisenstein, 2015).

Of course, some things are going to stay the same as we approach 2020. Expert microbial bioinformaticians are still primarily going to run command‐line programmes on the Linux operating system, typically using pipelines built from open‐source software glued together with homebrewed scripts, although these will be written in python rather than Perl (Myhrvold, 2014) or maybe in a yet‐to‐be‐devised scripting language. However, one should not rule out a role for commercial software packages, particularly for applications requiring accredited standard operating procedures. And, unfortunately, in 2020, there is still likely to be a dynamic tension between bioinformatics as an enabling and supporting technology for microbial genomics and bioinformatics as a scientific discipline in its own right, with consequent uncertainties reflected in the career structure and progression for microbial bioinformaticians (Pevzner, 2004; Watson, 2013).

As we approach the end of the current decade, there will be ever more microbial genomes and metagenomes and it remains uncertain whether databases and search strategies will be able to cope. Even in 2016, there is no easy way to download and search the metagenomic data accumulated by humankind, while BLAST searches of NCBI's supposedly non‐redundant database are beginning to strain under the weight of so many identical or near‐identical sequences from commonly sequenced species. And this is only going to get worse – for example, by 2020, we are going to have hundreds of thousands, if not millions of genome sequences from key bacterial species, such as *Escherichia coli* or *Mycobacterium tuberculosis*. New approaches to data storage and analysis are going to be required – for example, development of truly non‐redundant BLAST databases.

Those interested in microbial epidemiology and microbial population genetics, whether in the research or clinical context, are going to have to cope with the transition from systems based on a handful of gene sequences per organism [e.g. multilocus sequence typing (Maiden, 2006)] to whole‐genome approaches (Perez‐Losada *et al*., 2013; Ashton *et al*., 2016; Pankhurst *et al*., 2016). Some activities, such as manual curation and annotation of sequences or metadata by the individual enthusiast or by a dedicated research community, are just not sustainable during the scale‐up to the million‐microbial‐genome era. Instead, machine learning and artificial intelligence may have to fill the gap (Yip *et al*., 2013). And, sadly, the problem of lack of continuity of funding for databases and other bioinformatics resources is probably not going to be solved in the next few years (Parkhill *et al*., 2010).

After a period of lively competition (Loman *et al*., 2012), the marketplace for high‐throughput sequencing has recently settled into a state of near‐monopoly, with Illumina short‐read sequencing dominating the field. While this technology may be highly suited to applications such as re‐sequencing of genomes, where attention is focused on detection of single nucleotide variants, it is poorly able to cope with the riotous diversity of microbial genomes and metagenomes, particularly when looking at mobile genetic elements or accessory genomes (Stoesser *et al*., 2014). Single‐molecule long‐read technologies are already available at the time of writing in 2016 (e.g. from Pacific Biosystems or Oxford Nanopore), but are still waiting in the wings, despite progress in showing proof of principle applications (Loman *et al*., 2015; Quick *et al*., 2015, 2016) and developing bioinformatics tools dedicated to these approaches (Loman and Quinlan, 2014; Rhoads and Au, 2015; Watson *et al*., 2015). It remains unclear how far this will change in the coming years – will existing long‐read technologies take centre stage; or will new players enter the market? Whatever happens, both established and novel sequencing approaches are going to drive the development of new bioinformatics tools. Similarly, existing and new laboratory techniques focused on single‐cell genomics and transcriptomics (Lasken and McLean, 2014) or approaches to the functional genomics of microbes, such as RNA‐Seq (Creecy and Conway, 2015) or Tn‐Seq (Kwon *et al*., 2016), will create a continuing demand for new software.

Microbial genomics and metagenomics are hurtling full‐steam ahead into the clinical arena and into efforts to map the global landscape of microbial biodiversity (Pallen *et al*., 2010; Didelot *et al*., 2012; Robinson *et al*., 2013; Kyrpides *et al*., 2014; Brown *et al*., 2015; Luheshi *et al*., 2015; Spang *et al*., 2015). In both settings, it is clear that microbial taxonomy, with its polyphasic approach that requires laboratory culture and phenotypic investigation, is already broken and simply will not cope with an era in which most microorganisms are going to be identified and characterized through the analysis of macromolecular sequences (Chun and Rainey, 2014; Ramasamy *et al*., 2014; Thompson *et al*., 2015; Baltrus, 2016). Let us hope a new taxonomy is born by 2020, driven by – and driving – an explosion of creativity in the bioinformatics of microbial diversity (Varghese *et al*., 2015). Similarly, we can expect new opportunities and challenges arising from synthetic biology's desire to shift from merely reading to actively writing DNA sequences, whether in the creation of synthetic microorganisms or in novel approaches to data handling and storage (Goldman *et al*., 2013; Boeke *et al*., 2016; Hutchison *et al*., 2016).

The collision between microbial bioinformatics and human health care has already led to the development of new tools and this creative clash of disciplines is going to transform the outlook for bioinformaticians. Here, we are likely to see improvements in the tools for analysing microbial genomic epidemiology – for example, in tackling the growing realization that the cellular populations of pathogens, just like those of cancers, may well be clonal but that does not mean that they are necessarily homogeneous (Jamal‐Hanjani *et al*., 2015; Paterson *et al*., 2015). New models and new software will also need to recognize the problem of within‐host pathogen diversity and fact the pathogen phylogenies do not map simply on to transmission chains (Didelot *et al*., 2014; Gardy, 2016). But we hope that even if, as some have suggested, within‐host bacterial diversity makes it harder to reconstruct transmission networks, this will no longer pose a insuperable problem in 2020 (Worby *et al*., 2014).

Integration of microbial genomics and bioinformatics into clinical practice will bring fresh demands that pipelines not only be credible, robust and reproducible but produce easily interpretable clinician‐friendly outputs, for example, the programme Mykrobe, which analyses genomes from *Staphylococcus aureus* and *M. tuberculosis* (Bradley *et al*., 2015). Integration of sequence data with clinical metadata will be difficult, particularly as precision medicine is going to need precise ontologies (Dugan *et al*., 2014) – for example, in analysing a hospital outbreak, the next generation of NHS bioinformaticians are going to have to be highly attuned to, say, the difference between a ‘bed’ and a ‘bed space’. They will be assisted in these efforts as the ‘internet of things’ penetrates healthcare systems, so that a patient, an instrument or even a piece of hospital furniture or plumbing will have its own IP address and GPS‐savvy chip and all providing information that can be integrated with pathogen genome sequences (Hao and Wang, 2015).

Metagenomics as a diagnostic approach is likely to move closer to routine practice (Loman *et al*., 2013; Doughty *et al*., 2014; Pallen, 2014; Wilson *et al*., 2014), but reliably disentangling pathogen genomes from metagenomes – particularly if short‐read technologies still dominate the field – is going to present a formidable challenge (Alneberg *et al*., 2014).

The current mania for microbiomes looks set to continue, so new bioinformatics tools to detect ‘sick microbiomes’ and link them to disease states are going to be required (Forslund *et al*., 2015). Perhaps by 2020, the tide will have turned away from molecular barcoding approaches, epitomized by what has been called the one‐eyed king (Forney *et al*., 2004), 16S ribosomal RNA gene sequences, towards the more widespread adoption of shotgun metagenomics (Jovel *et al*., 2016). If so, new tools will be required to translate metagenomes into the standard outputs of microbial ecology (rarefaction curves, diversity indices etc.). Similarly, new tools will emerge at the interface between metagenomics, metatranscriptomics, metabolomics and systems biology (Franzosa *et al*., 2014).

A potential concern is the growth of a wild frontier of microbial genome and microbiome analyses performed by non‐experts, hand‐cranking data through pipelines that they do not fully understand and then naively interpreting results without engaging the healthy scepticism of the seasoned expert (Bhatt *et al*., 2013; Branton *et al*., 2013; Laurence *et al*., 2014; Salter *et al*., 2014; Strong *et al*., 2014; Ackelsberg *et al*., 2015; Afshinnekoo *et al*., 2015). Eternal vigilance is likely to be the price of containing the equivalent of microbial genomic astrology!

When it comes to provisioning of hardware and software, microbial bioinformatics is being pulled away from the archetypal self‐administered server or cluster run by a single‐user or single‐research‐group. In one direction lies the development of apps for mobile devices (Rose *et al*., 2013; Wong *et al*., 2013; Nguyen *et al*., 2014), in parallel with the rise of palmtop sequencing (Quick *et al*., 2016), so that in 2020, sequencing and analysis may well take place out in the field and/or much closer to the patient. The centralizing efforts of national or transnational projects are pulling in another direction, aimed at standardizing protocols for creating, storing and analysing microbial sequence data, particularly for healthcare, although it perhaps unlikely that such efforts will have arrived at a stable agreed global solution by 2020 (Moran‐Gilad *et al*., 2015).

Another potential trend is the rise of crowd‐sourced microbial bioinformatics analyses, performed by bioinformaticians around the globe – there have already been proof‐of‐principle cases (Rohde *et al*., 2011; Gardy *et al*., 2015) and we are likely to see more of this by 2020, particularly in response to public‐health emergencies. Similarly, microbial bioinformaticians are likely to embrace cloud computing (Drake, 2014), which brings economies of scale in effort and costs and liberates end users from the hassle of maintaining systems and setting up commonly used software, while also delivering improvements in the sharing of pipelines and data, which in turn will enhance the reproducibility of bioinformatics analyses. One promising example here is the UK's Cloud Infrastruture for Microbial Bioinformatics (CLIMB) project, which provides end users in the microbiology community with access to virtual machines provided via the OpenStack open‐source cloud‐computing environment (Connor *et al.,* 2016).

One final challenge for microbial bioinformatics in the run‐up to 2020 is meeting the need for training and the development of a skilled labour force (Via *et al*., 2013; Watson‐Haigh *et al*., 2013). Cloud computing may play a contribution here, in providing a standardized environment for workshops and hackathons as well as for research groups. Similarly, we can expect to see a continued rise in open‐source training materials suitable for use in bioinformatics boot camps and the development of new workflow and data integration systems, such as the Genomics Virtual Laboratory (Afgan *et al*., 2015).

In conclusion, microbial bioinformatics in 2020 will remain a vibrant, creative discipline, adding value to the ever‐growing flood of new sequence data while embracing new technologies and new approaches. As we boldly go into the third decade of the 21st century, microbial sequence space will remain the final frontier!

## Conflict of interest

None declared.
